# Physical Fitness Differences, Amenable to Hypoxia-Driven and Sarcopenia Pathophysiology, between Sleep Apnea and COVID-19

**DOI:** 10.3390/ijerph19020669

**Published:** 2022-01-07

**Authors:** Vasileios T. Stavrou, George D. Vavougios, Stylianos Boutlas, Konstantinos N. Tourlakopoulos, Eirini Papayianni, Kyriaki Astara, Ilias T. Stavrou, Zoe Daniil, Konstantinos I. Gourgoulianis

**Affiliations:** Laboratory of Cardio-Pulmonary Testing and Pulmonary Rehabilitation, Department of Respiratory Medicine, Faculty of Medicine, University of Thessaly, Biopolis, 4100 Larissa, Greece; dantevavougios@hotmail.com (G.D.V.); sboutlas@gmail.com (S.B.); kostisst@hotmail.com (K.N.T.); eirinipapayianni@gmail.com (E.P.); kyriakiastara@gmail.com (K.A.); iliasssstavrou@gmail.com (I.T.S.); zdaniil@uth.gr (Z.D.); kgourg@uth.gr (K.I.G.)

**Keywords:** handgrip, fitness, muscle mass, fatigue, body composition

## Abstract

Handgrip strength is an indirect indicator of physical fitness that is used in medical rehabilitation for its potential prognostic value. An increasing number of studies indicate that COVID-19 survivors experience impaired physical fitness for months following hospitalization. The aim of our study was to assess physical fitness indicator differences with another prevalent and hypoxia-driven disease, Obstructive Sleep Apnea Syndrome (OSAS). Our findings showed differences between post-COVID-19 and OSAS groups in cardiovascular responses, with post-COVID-19 patients exhibiting higher values for heart rate and in mean arterial blood pressure. Oxygen saturation (SpO_2_) was lower in post-COVID-19 patients during a six-minute walking test (6MWT), whereas the ΔSpO_2_ (the difference between the baseline to end of the 6MWT) was higher compared to OSAS patients. In patients of both groups, statistically significant correlations were detected between handgrip strength and distance during the 6MWT, anthropometric characteristics, and body composition parameters. In our study, COVID-19 survivors demonstrated a long-term reduction in muscle strength compared to OSAS patients. Lower handgrip strength has been independently associated with a prior COVID-19 hospitalization. The differences in muscle strength and oxygenation could be attributed to the abrupt onset of the disorder, which does not allow compensatory mechanisms to act effectively. Targeted rehabilitation focusing on such residual impairments may thus be indispensable within the setting of post-COVID-19 syndrome.

## 1. Introduction

COVID-19 has led to an increase in morbidity worldwide. Due to secondary respiratory failure both in the hospital and in post-COVID-19 settings, patients limit their physical activities, particularly when experiencing severe disease, as desaturation occurs even during minimal mobilization. Exposure to hypoxia and limitations in physical activity are shared features of Obstructive Sleep Apnea Syndrome (OSAS) and post-COVID-19 syndrome [1,2,3]. Bedridden patients experience functional decline and an increased risk of complications proportionately to their hospitalization time and regardless of age [4]. Even one week of hospitalization affects muscle mass and the patient’s general wellness [5]. An indirect measure of patient fitness is handgrip strength [6], with prognostic utility regarding physical ability and cardiovascular status in hospitalized patients [7].

The aim of this study was to investigate physical fitness indicator differences between patients with OSAS and COVID-19, as two distinct hypoxia-driven diseases. We hypothesized that these diseases share pathophysiologic commonalities in the context of respiratory and musculoskeletal function, which could offer insights in optimizing the management of these diseases.

## 2. Materials and Methods

### 2.1. Study Population

The study population included adult COVID-19 survivors that had previously been hospitalized in the Respiratory Department of the University Hospital of Larisa (*n* = 40), versus obstructive OSAS patients (*n* = 40) without a history of or active infection with COVID-19. Patients were recruited between September 2020 to June 2021 (total *n* = 80; see Table 1 for cohort characteristics).

### 2.2. Inclusion and Exclusion Criteria

The following inclusion criteria were applied: (i) patients no longer requiring supplemental O_2_, (ii) patients who were fever-free for a period of at least 48 h (without medication to reduce the fever) prior to enrollment and/or (iii) patients deemed to be stable according to the National Institute of Health and Hellenic guidance criteria for COVID-19 discharge [8], and (iv) patients with at least 60 days between discharge and enrollment. The inclusion criteria for OSAS patients were the following: (i) presenting with Apnea Hypopnea Index ≥ 15 events/h, (ii) nonsmokers, aged between ≥30 and ≤60 years old, and (iii) sleep duration ≥ 300 min during polysomnography study (PSG) [9]. The exclusion criteria, for both groups, were contraindications for 6MWT [9,10], body mass index ≥40 kg/m^2^, abnormal pulmonary function test (FEV_1_ ≤ 85%, FEV_1_/FVC < 80, and DLCO < 80%), and musculoskeletal disability which could impair maximum exercise capacity [9,11]. Moreover, post-COVID-19 patients with Pittsburgh Sleep Quality Index score ≤ 5 [12] and Epworth Sleep Scale score < 10 [13] were also excluded.

### 2.3. Study Ethics

The study’s protocol was approved by the Institutional Review Board (IRB)/Ethics Committee (EC) of the University Hospital of Larissa (IRB/EC approval reference number: 15314/21-04-2021). All participants provided written informed consent, in accordance with the Helsinki declaration and personal data protection requirements under the European Parliament and the Council of the European Union legislation [14].

### 2.4. Measurements

Prior to conducting physical fitness tests, the medical history, anthropometric characteristics (i.e., body height (Seca 700, Hamburg, Germany), chest circumference in maximal inhalation and exhalation (Δchest), neck circumference, and waist–hip ratio (Seca 201, Hamburg, Germany)), and body composition (i.e., body mass, muscle mass, percentage of body fat, visceral fat score, lean body mass and total body water (Tanita MC-980, Arlington Heights, IL, USA)) [9] of patients were recorded.

### 2.5. Physical Fitness Tests

The 6-minute walk test (6MWT) was performed according to the ATS guidelines [15] with O_2_ saturation (SpO_2_) and heart rate (HR) (Nonin 9590 Onyx Vantage, Plymouth, MN, USA) having been recorded at baseline and every one minute of the test thereafter [8,9]. Blood pressure (Sphygmomanometer Mac Check 501, Tokyo, Japan) and self-assessment for dyspnea and lower limbs fatigue [16] were recorded before and at the end of the test. Finally, the total distance during the 6MWT was used to estimate the oxygen uptake [17] according to the equation: [O_2_ uptake = 4.948 + 0.023 × distance _(m)_].

Handgrip strength was assessed with a standard height chair (46 cm), with the patient’s elbow being by the side of the body and flexed at 90 degrees; the forearm and wrist remained in a neutral position and the handle of the electronic dynamometer (Camry, EH 101, South El Monte, CA, USA) rested on the middle of the four fingers [18]. All participants performed one maximum isometric effort for 5 s with the dominant hand. All subjects reported their dominant upper limb before the trials.

For the physical fitness test, a demonstration was performed by the clinical exercise physiologist prior to the patients’ own attempts. All patients performed the physical fitness tests without encouragement, in the Laboratory of Cardio-Pulmonary Testing and Pulmonary Rehabilitation (University of Thessaly), with the environmental temperature at 24.1 ± 2.1 °C and humidity 33.8 ± 4.2%. The evaluation of patients was performed between 09:30 a.m. and 13:30 p.m.

### 2.6. Statistical Analysis

Data are presented as mean ± SD and frequency (%) where appropriate. Data normality was assessed using the Kolmogorov–Smirnov One-Sample test. The independent samples *t*-test was used to assess differences between groups (post-COVID-19 patients vs. the OSAS group). Pearson correlation analysis was used for statistical comparison between variables. Multivariate analyses were performed via Backward Stepwise Logistic Regression (BSLR). Input variables included handgrip, gender, age, body mass index, body surface area, lean body mass, total body water, body fat, muscle mass, visceral fat, waist–hip ratio, Δchest, distance walked during the 6MWT, dyspnea at the end of the 6MWT, estimated oxygen uptake (*V*O_2peak_), HR, and SpO_2_ in the sixth minute of the 6MWT. For all tests, a *p*-value < 0.05 was considered statistically significant. Continuous variables were reported as mean values with standard deviation (mean ± SD). The IBM SPSS 21 statistical package (SPSS Inc., Chicago, IL, USA) was used for all statistical analyses.

## 3. Results

Table 1 presents anthropometric characteristics and body composition results between the groups. Table 2 presents the results of the 6MWT, divided between the groups. Handgrip strength test showed lower values in post-COVID-19 patients (39.2 ± 10.3 vs. 44.0 ± 11.0 kg, t_(78)_ = −2.037, *p* = 0.045) compared to the OSAS group.

**Figure 1:**
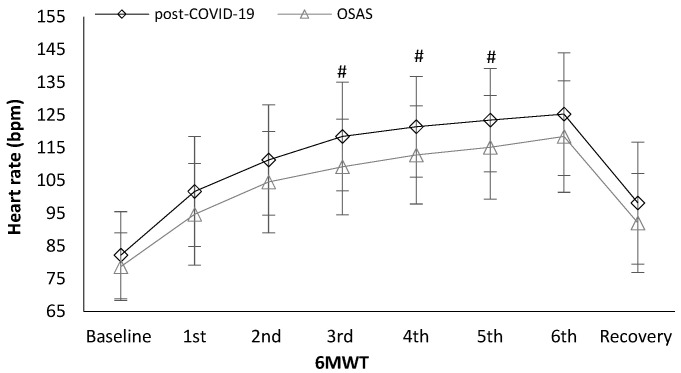
Heart rate alteration during the 6-minute walk test (6MWT) between the groups. # < 0.005.

**Figure 2:**
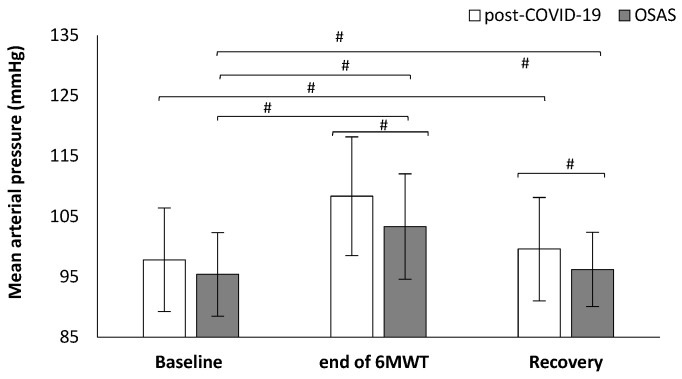
Mean arterial pressure alteration during the 6-minute walk test (6MWT) between the groups. # < 0.005.

Heart rate was significantly different between the groups. Post-COVID-19 patients showed higher values in heart rate in the third minute (118.5 ± 16.6 vs. 109.2 ± 14.6 bpm, t_(78)_ = 2.659, *p* = 0.010, Figure 1), fourth minute (121.4 ± 15.4 vs. 112.8 ± 15.0 bpm, t_(77)_ = 2.519, *p* = 0.014, Figure 1), and fifth minute (123.5 ± 15.8 vs. 115.1 ± 15.8 bpm, t_(77)_ = 2.345, *p* = 0.022, Figure 1) during the 6MWT compared to the OSAS group. Post-COVID-19 patients had higher values in mean arterial blood pressure at the end of the 6MWT (108.4 ± 9.8 vs. 103.4 ± 8.7 mmHg, t_(78)_ = 2.418, *p* = 0.018, Figure 2) and in the first minute of recovery (99.6 ± 8.6 vs. 96.2 ± 6.2 mmHg, t_(78)_ = 2.023, *p* = 0.046, Figure 2) compared to the OSAS group.

**Figure 3:**
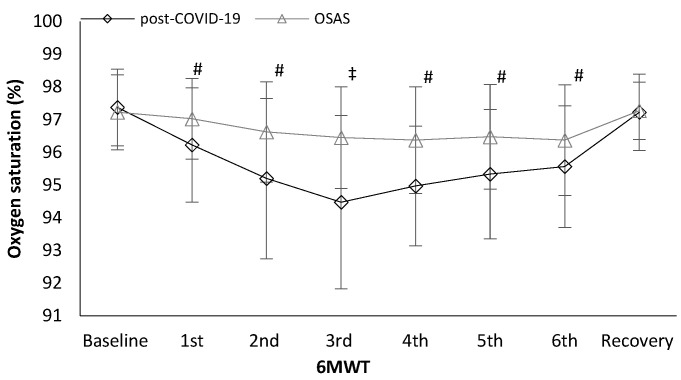
Oxygen saturation alteration during the 6 min walk (6MWT) test between the groups. # <0.005, *‡* <0.001.

Oxygen saturation was lower in post-COVID-19 patients in the first minute (96.2 ± 1.7 vs. 97.0 ± 1.2%, t_(78)_ = −2.369, *p* = 0.020, Figure 3), second minute (95.2 ± 2.5 vs. 96.6 ± 1.5%, t_(78)_ = −3.118, *p* = 0.003, Figure 3), third minute (94.5 ± 2.7 vs. 96.5 ± 1.6 %, t_(78)_ = −4.067, *p* < 0.001, Figure 3), fourth minute (94.7 ± 1.8 vs. 96.4 ± 1.6 %, t_(77)_ = −3.599, *p* = 0.001, Figure 3), fifth minute (95.3 ± 1.9 vs. 96.5 ± 1.6 %, t_(77)_ = −2.823, *p* = 0.006, Figure 3), and sixth minute (95.6 ± 1.9 vs. 96.4 ± 1.7 %, t_(77)_ = −2.029, *p* = 0.046, Figure 3) during the 6MWT compared to the OSAS group. Differences in oxygen saturation (ΔSpO_2_; baseline to end of the 6MWT) were higher in post-COVID-19 patients (2.2 ± 2.1 vs. 1.1 ± 1.2 %, t_(78)_ = 2.707, *p* = 0.008) compared to in OSAS patients.

**Figure 4:**
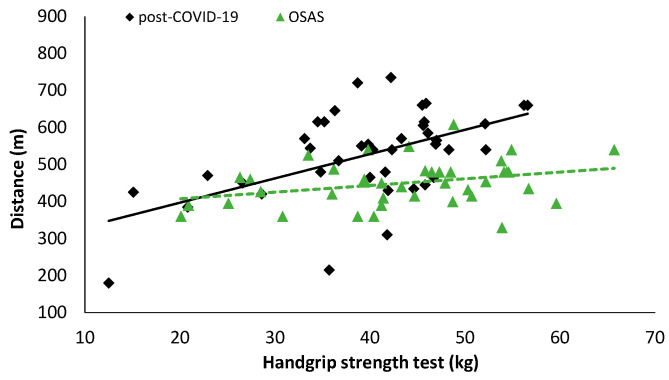
Correlation analysis results between the handgrip strength test and distance during the 6MWT (post-COVID-19: r = 0.540, *p* < 0.001; OSAS: r = O.322, *p* = 0.044).

**Figure 5:**
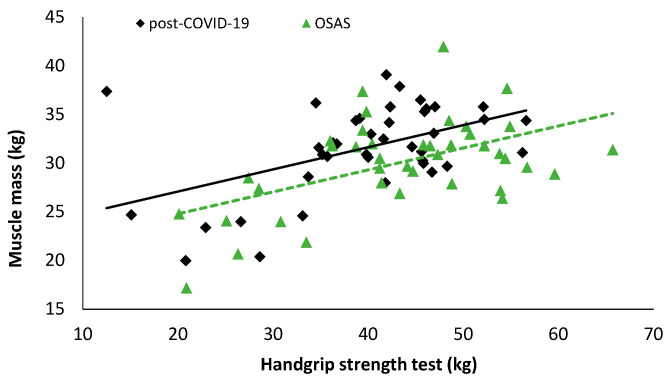
Correlation analysis results between handgrip strength test and muscle mass (post-COVID-19: r = 0.492, *p* = 0.001; OSAS: r = 0.522, *p* = 0.001).

In patients with OSAS, statistically significant correlations were detected between handgrip and distance during the 6MWT (r = 0.322, *p* = 0.044, Figure 4), anthropometric characteristics, and body composition parameters (muscle mass: r = 0.522, Figure 5, *p* = 0.001; body mass: r = 0.370, *p* = 0.019; lean body mass: r = 0.522, *p* = 0.001; percent of body fat: r = −0.499, *p* = 0.001; total body water: r = 0.672, *p* < 0.001; neck circumference: r = 0.474, *p* = 0.002); and estimated O_2_ uptake (r = 0.325, *p* = 0.041). In post-COVID-19 patients, statistically significant correlations were detected between handgrip and distance covered during the 6MWT (r = 0.540, *p* < 0.001, Figure 4), anthropometric characteristics, and body composition parameters (muscle mass: r = 0.492, *p* = 0.001 Figure 5; body mass: r = 0.508, *p* = 0.001; lean body mass: r = 0.634, *p* < 0.001; percent of body fat: r = −0.457, *p* = 0.003; total body water: r = 0.572, *p* < 0.001; visceral fat: r = 0.429, *p* = 0.006; WHR: r = 0.465, *p* = 0.002; Δchest: r = 0.379, *p* = 0.016); O_2_ saturation at the end of the 6MWT (6th minute: r = −0.333, *p* = 0.039); and estimated O_2_ uptake (r = 0.538, *p* < 0.001).

In patients with OSAS, statistically significant correlations were detected between the distance covered during the 6MWT and leg fatigue before the 6MWT (r = −0.407, *p* = 0.009), heart rate during the 6MWT (4th minute: r = 0.344, *p* = 0.030; 5th minute: r = 0.400, *p* = 0.011; 6th minute: r = 0.349, *p* = 0.027), differences in heart rate (baseline to end of the 6MWT: r = 0.416, *p* = 0.008), and O_2_ saturation during the 6MWT (2nd minute: r = 0.384, *p* = 0.015; 4th minute: r = 0.315, *p* = 0.048). In post-COVID-19 patients, statistically significant correlations were detected between the 6MWT and muscle mass (r = 0.317, *p* = 0.046), Δchest (r = 0.333, *p* = 0.036), and leg fatigue at the end of the 6MWT (r = −0.362, *p* = 0.022), dyspnea in the baseline (r = −0.478, *p* = 0.002) and at the end of the 6MWT (r = −0.375, *p* = 0.017), and O_2_ saturation during the 6MWT (4th minute: r = −0.390, *p* = 0.014; 5th minute: r = −0.319, *p* = 0.048).

The results of multivariate analyses via BSLR are presented in Table 3.

## 4. Discussion

**Figure 6:**
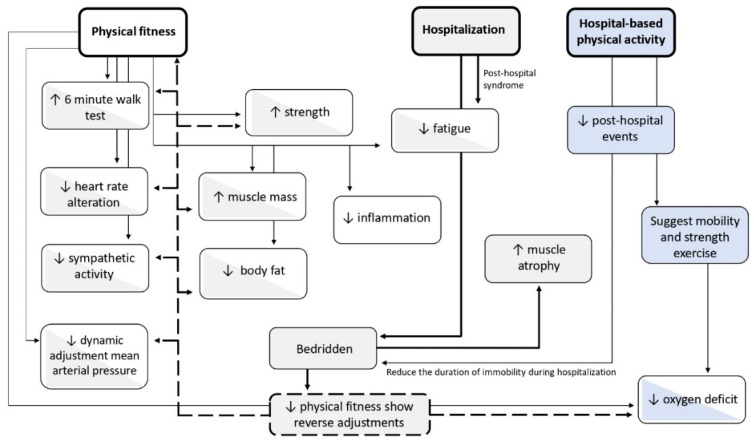
Physiological adaptations of exercise and reduced exercise in healthy adults (white boxes), hospitalized patients (grey boxes), and physical activity during hospitalization patients (blue boxes) and bicolor boxes show the reverse adjustments. Dotted lines: reverse, detrimental adjustments in physical fitness indicators due to hospitalization and/or long time bedridden/immobility. Continuous lines: adjustments during exercise (mild lines) and during hospitalization (bold lines).

In our study, we aimed to assess differences in handgrip strength, an indirect measure of muscle strength, between OSAS patients and COVID-19 survivors. Our findings indicated that COVID-19 survivors (at a two month post-discharge timepoint) have reduced muscle strength compared to OSAS patients. Furthermore, we reported an independent association between lower handgrip strength and prior hospitalization due to COVID-19 (Figure 6).

### 4.1. Hypoxia and Reduced Strength in COVID-19 Survivors and OSAS Patients

Our findings indicate that COVID-19 survivors present with lower oxygen saturation during mobilization compared to OSAS patients, as indicated by measurements during the 6MWT. Hypoxemia in post-COVID-19 patients has been attributed to ventilation-to-perfusion (*V*/*Q*) mismatch in scattered lung units, especially in the early stages [19]. The *V*/*Q* ratio may be minimized in certain patients, as ventilation is hindered due to the lungs’ low elastance and recruitability secondary to structural lung damage, whereas in others the *V*/*Q* ratio is elevated due to the superiority of ventilated non perfused lung units over non ventilated ones (shunt) [3]. As far as perfusion is concerned, microvascular thrombosis has been well-documented to occur in COVID-19 patients [20]. The implication of this process in the context of the lung represents dead space with reduced/absent pulmonary capillary flow without affecting ventilation, leading to a high *V*/*Q* ratio. Additionally, in the early stages of infection the inflammation affects the lungs in a nonuniform manner, leading to an uneven distribution of capillary perfusion units [21]. This process leads some alveolocapillary units to higher *V*/*Q* ratios. While COVID-19 survivors are considered disease-free regarding the acute phase [22], persistent oxygen desaturation and fatigue are commonly reported in the post-acute period.

Conversely, chronic hypoxia in OSAS is characterized by intermittent hypoxia that is state-dependent—i.e., during sleep [23]. Intermittent hypoxia is characterized by cycles of hypoxemia with reoxygenation and is recognized as a potential fundamental factor contributing to the pathogenesis of OSAS-related comorbidities [24]. According to Dewan et al. [23], “OSAS patients, typically manifest short intermittent high-frequency hypoxemic episodes occurring in an approximately 8 h interval of sleep while, sustained or low-frequency hypoxemia with SaO_2_ ranging between 80–85% that lasts longer, from a few minutes to hours, is witnessed primarily during rapid ascent and descent from altitude and secondary in chronic respiratory disease during sleep”. The major pathophysiologic difference between the short intermittent high-frequency hypoxemia, as seen in OSAS, and sustained prolonged low-frequency hypoxemia is the cycles of reoxygenation. Intermittent hypoxia promotes increased systemic and vascular inflammation with endothelial dysfunction and sympathetic excitation during both daytime and nighttime and contributes to multiorgan comorbidity [23]. Comparing COVID-19 survivors and OSAS, we account for the differences in muscle strength and oxygenation in the abrupt onset of the disorder, which does not allow compensatory mechanisms to take action effectively.

It is worth noting that obesity was prevalent in both groups, and it is a well-known risk factor for both entities [25,26,27]. In OSAS patients, obesity contributes to *V*/*Q* mismatch by impairing ventilation, as it reduces lung compliance and volumes, decreases respiratory muscle strength and increases airway resistance. Reduced ventilation promotes a hypercapnic condition [28]. From our results, body fat percentage and neck circumference—as more accurate determinants—were substantially high in both groups. Furthermore, we may attribute their discrepancies to their state—dependence differences as COVID-19 is an acute debilitating condition with the patient becoming dehydrated, malnourished, and cachectic under the weight of the disease. Therefore, their overall weight rapidly reduces due to the loss of fluids and lean muscle mass.

### 4.2. Reduced Handgrip Strength as a Consequence of Loss of Muscle Mass

Sarcopenia is a complex syndrome composed of nutritional deficiency, chronic inflammation, insulin resistance, and a decline in anabolic hormones and is directly related to reductions in mobility and functional status. Prolonged periods of bed rest (>10 days) have been shown to induce substantial changes in body composition and are accompanied by overall metabolic decline [29]. Additionally, bed rest can affect potentially all body muscles, particularly postural ones, such as rectus abdominis, external oblique, internal oblique, transversus abdominis, thoracic erector spinae, as well as the lumbar multifidus muscle, vastus lateralis, and soleus [5]. Correspondingly, lean body mass and the quadriceps cross-sectional area may also be affected by a loss of significant thickness and peak oxygen uptake (*V*O_2peak_) may also be affected, which increases the overall risk of falls and disability [30]. Severe sarcopenia refers to the most debilitating evolution of this syndrome that prevents individuals from accomplishing the simple activities of daily living, leaving them unable to self-care [31]. COVID-19 survivors may experience acute sarcopenia [32], preceded by lockdown-associated sarcopenia [33]. This connection carries over into post-COVID-19 syndrome [34]. These associations are reflected in our results and should be addressed by targeted rehabilitation.

## 5. Conclusions

Our findings suggest that COVID-19 survivors display diminished muscle strength compared to OSAS patients, as measured by handgrip. Lower handgrip strength was independently associated with a risk of COVID-19 hospitalization, and, considering its correlates, may be an important target for rehabilitation. Specifically, as COVID-19 survivors experience hypoxia during physical mobilization, indicating a combination of impaired respiratory function and sarcopenia, these residual impairments should be identified and reversed via targeted approaches.

## Figures and Tables

**Table 1:** Participants’ characteristics. Data are expressed as mean ± standard deviation.

	Post-COVID-19	OSAS	*p*-Value
Age, yrs	51.7 ± 6.5	48.3 ± 9.5	0.072
Gender, F/M	7/33	7/33	-
Body mass, kg	90.2 ± 13.0	101.9 ± 19.9	0.002
Body mass index, kg/m^2^	29.7 ± 4.3	33.2 ± 6.3	0.005
Body surface area, m^2^	2.2 ± 0.4	2.5 ± 0.5	0.004
Body fat, %	30.3 ± 9.0	34.0 ± 9.1	0.069
Muscle mass, kg	31.5 ± 4.6	30.1 ± 4.7	0.155
Visceral fat, score	12.5 ± 3.7	15.0 ± 5.6	0.024
Lean body mass, kg	64.1 ± 6.5	69.1 ± 9.1	0.006
Total body water, %	48.2 ± 9.1	46.9 ± 8.8	0.529
Neck circumference, cm	40.9 ± 9.3	41.1 ± 4.2	0.914
Waist–hip ratio	1.0 ± 0.1	1.0 ± 0.1	0.416
Δchest	6.6 ± 3.0	6.3 ± 1.7	0.676

Abbreviations: Δchest: the difference in chest circumference between maximal inhalation and exhalation.

**Table 2:** 6MWT results between groups. Data are expressed as mean ± standard deviation.

	Post-COVID-19	OSAS	*p*-Value
Distance, m	525.6 ± 120.6	449.7 ± 61.0	0.001
Distance, % of predicted	88.0 ± 18.6	76.5 ± 12.9	0.002
Estimated *V*O_2peak_, ml/min/kg	17.0 ± 2.8	15.3 ± 1.4	0.001
Metabolic equivalent	4.9 ± 0.8	4.4 ± 0.4	0.001
Leg Fatigue, Borg scale			
Baseline	0.4 ± 0.8	0.8 ± 0.9	0.085
End of 6MWT	1.3 ± 1.3	1.3 ± 1.2	0.928
Dyspnea, Borg scale			
Baseline	0.5 ± 0.9	0.9 ± 1.1	0.108
End of 6MWT	1.6 ± 1.7	1.4 ± 1.3	0.607

Abbreviations: 6MWT: 6-minute walk test; *V*O_2peak_: oxygen uptake in the maximal effort.

**Table 3:** Multivariate analyses via backwards stepwise logistic regression BSLR revealed several independent associations with COVID-19.

							95% C.I.for EXP(B)	
	B	S.E.	Wald	df	Sig.	Exp(B)	Lower	Upper
Handgrip, kg	−0.135	0.039	12.114	1	0.001	0.874	0.810	0.943
Body fat, %	−0.077	0.034	5.001	1	0.025	0.926	0.866	0.991
METs	2.496	0.640	15.205	1	0.000	12.135	3.461	45.552

## Data Availability

All data are available upon request.
